# Methylation as a critical epigenetic process during tumor progressions among Iranian population: an overview

**DOI:** 10.1186/s41021-021-00187-1

**Published:** 2021-04-21

**Authors:** Iman Akhlaghipour, Amir Reza Bina, Mohammad Reza Abbaszadegan, Meysam Moghbeli

**Affiliations:** 1 Student Research Committee, Faculty of Medicine, Mashhad University of Medical Sciences, Mashhad, Iran; 2 Student Research Committee, Faculty of Medicine, Birjand University of Medical Sciences, Birjand, Iran; 3 Medical Genetics Research Center, Mashhad University of Medical Sciences, Mashhad, Iran; 4 Department of Medical Genetics and Molecular Medicine, School of Medicine, Mashhad University of Medical Sciences, Mashhad, Iran

**Keywords:** Diagnostic panel, Epigenetic, Methylation, Early detection, Cancer, Iran

## Abstract

Cancer is one of the main health challenges and leading causes of deaths in the world. Various environmental and genetic risk factors are associated with tumorigenesis. Epigenetic deregulations are also important risk factors during tumor progression which are reversible transcriptional alterations without any genomic changes. Various mechanisms are involved in epigenetic regulations such as DNA methylation, chromatin modifications, and noncoding RNAs. Cancer incidence and mortality have a growing trend during last decades among Iranian population which are significantly related to the late diagnosis. Therefore, it is required to prepare efficient molecular diagnostic panels for the early detection of cancer in this population. Promoter hyper methylation is frequently observed as an inhibitory molecular mechanism in various genes associated with DNA repair, cell cycle regulation, and apoptosis during tumor progression. Since aberrant promoter methylations have critical roles in early stages of neoplastic transformations, in present review we have summarized all of the aberrant methylations which have been reported during tumor progression among Iranian cancer patients. Aberrant promoter methylations are targetable and prepare novel therapeutic options for the personalized medicine in cancer patients. This review paves the way to introduce a non-invasive methylation specific panel of diagnostic markers for the early detection of cancer among Iranians.

## Background

**Fig. 1:**
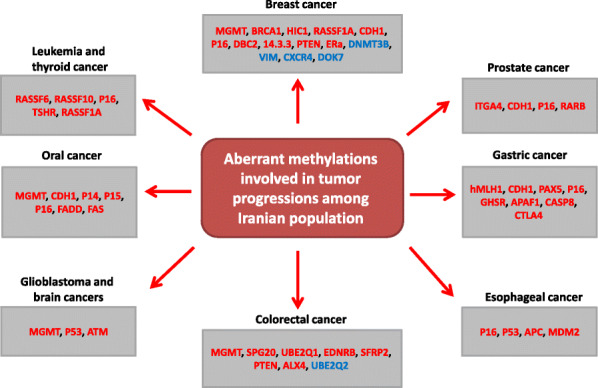
all of the aberrant methylations involved in tumor progression among Iranian population. Blue and red colors refer to the hypo and hypermethylation, respectively

Cancer is the main and second cause of death in developed and developing countries, respectively [1]. It is the third most common cause of death among Iranian population [2]. Gastric and breast cancers are the most common malignancies among Iranian men and women, respectively [3]. Lifestyle and environmental changes were occurred during the recent years due to the rapid industrialization in Iran [1]. Various environmental risk factors including tobacco smoking, environmental chemicals, high dietary salt intake, bacterial and viral infections, and gastro-esophageal reflux have been reported for cancer among Iranians [4–6]. Epigenetic involves the heritable and reversible transcriptional changes without any DNA sequence alterations which are involved in the early stages of tumor progression, embryogenesis, imprinting, and X-chromosome inactivation [7, 8]. It is regulated via different processes such as DNA methylation, chromatin modifications, and noncoding RNAs that play critical roles during tumor initiation and progression [9–11]. DNA methylation involves the transfer of a methyl group to the cytosine that is catalyzed by DNA methyltransferase (DNMT) [12, 13]. DNMT inhibitors are categorized into the nucleoside analogs and the non-nucleoside inhibitors [14]. The azacytidine and decitabine as nucleoside analogs are the most common DNMT inhibitors and epigenetic modulators in cancer therapy [15]. Non-nucleoside compounds such as hydralazine and procainamide inhibit the methylation through a DNA incorporation independent mechanism [14]. Curcumin belongs to the Non-nucleoside DNMT inhibitors that bind with DNMT1 catalytic domain [16]. DNA hypo methylation leads to aberrant activation of oncogenes while the hyper methylation is associated with inhibition of tumor suppressor genes. Various tumor suppressor genes such as p16, MutL homolog 1 (MLH1), and breast cancer type 1 susceptibility protein (BRCA1) which are involved in DNA repair, cell cycle, cell adhesion, and apoptosis have been shown to undergo tumor-specific silencing by hyper methylation [17–19]. Histone modifications through histone acetyl-transferase (HATs), histone methyltransferase (HMTs), kinases, ubiquitin ligases, and sumoligases are important regulatory processes in chromatin remodeling, gene expression, and carcinogenesis [20, 21]. Micro RNAs are also the post transcriptional regulators of more than 60% of protein-coding genes during various cellular processes that can be associated with tumorigenesis [22, 23]. Epigenetic markers are considered as emerging diagnostic and prognostic biomarkers in cancer [24, 25]. Since, aberrant DNA methylation can be tracked in body fluids; they can be suggested as efficient diagnostic and prognostic markers in primary stages of tumor progression [26–29]. It has been reported that the majority of cancer related deaths among Iranian cases are associated with late diagnosis. Therefore, it is required to determine novel diagnostic markers for the early detection of cancer in this population. In present review we have summarized all of the significant epigenetic deregulations associated with tumor progression which have been reported until now among Iranian cancer patients (Fig. 1) (Table 1).

**Table 1:** all of the aberrant methylations which have been reported among Iranian cancer patients

Gene	Year	Type	population	sample	Results	study (ET AL)
dna repair						
MGMT, CDH1	2010	Oral	57N/76T^a^	Tissue	Hyper methylation.	Kordi-Tamandan [30]
MGMT, BRCA-1	2019	Breast	27 MT^b^ 31 BT^c^	Tissue	Hyper methylation in MT.	Paydar [31]
MGMT, p53	2009	Glioblastoma	50 patients	Tissue	MGMT hyper methylation was correlated with p53 expression.	Shamsara [32]
MGMT	2013	Colorectal	40 patients 30 controls	Tissue	Hyper methylation.	Farzanehfar [33]
MGMT	2018	Colorectal	70 patients	Serum	Hyper methylation.	Alizadeh Naini [34]
hMLH1, CDH1	2014	Gastric	51 patients	Tissue	Hyper methylations were correlated with stage.	Moghbeli [35]
cell adhesion						
RASSF6, RASSF10	2017	Lymphoblastic leukemia	45 patients	Blood	Hyper methylation.	Younesian [36]
HIC1,RASSF1A	2009	Breast	81 patients 100 controls	Tissue	Hyper methylation.	Rasti [37]
P16, TSHR, RASSF1A,	2011	Thyroid	25MT 25BT	Tissue	Hyper methylation.	Mohammadi-asl [38]
Integrin *a*4, CDH1	2015	Prostate	30 patients 40 benign	Tissue	Hyper methylation.	Mostafavi-Pour [39]
CDH1	2016	Breast	98T/10N	Tissue	Hyper methylation.	Naghitorabi [40]
CDH1	2014	Breast	50 N/T	Tissue	Hyper methylation.	Shargh [41]
SPG20	2017	Colorectal	32 N/T	Serum	Hyper methylation.	Rezvani [42]
cell cycle						
P14ARF	2010	Oral	76 patients 57 controls	Tissue	Hyper methylation.	Kordi-Tamandani [43]
P16INK4a, p53, p16, MDM2	2010	Esophageal	50 N/T	Tissue	P16 hyper methylation was correlated with p53 expression.	Taghavi [44]
P16	2005	Esophageal	58 patients 30 controls	Blood	Hyper methylation.	Abbaszadegan [45]
P16	2018	Oral	67 patients 59 controls	Tissue	Hyper methylation was correlated with grade.	Allameh [46]
P15INK4a, p16INK4a	2012	Oral		Tissue	Hyper methylation.	Kordi-Tamandani [47]
P16	2009	Breast	70 patients		Hyper methylation.	Vallian [48]
P16	2008	Gastric	52 patients 50 controls	Serum	Hyper methylation.	Abbaszadegan [49]
DBC2	2012	Breast	50 patients 35 controls	Tissue	Hyper methylation.	Hajikhan Mirzaei [50]
14-3-3 sigma	2012	Breast	20 patients 20 controls	Tissue	Hyper methylation.	Gheibi [51]
UBE2Q1, UBE2Q2	2015	Colorectal	60 N/T 20 BT	Tissue	UBE2Q2 hypo methylation, UBE2Q1 hyper methylation.	Mokarram [52]
tyrosine kinases and g protein coupled receptors						
VIM, CXCR4, DOK7	2018	Breast	60 patients 40 controls	Blood	Hypo methylation.	Shirkavand [53]
GHSR	2019	Gastric	22 N/T	Tissue	Hyper methylation.	Amini [54]
EDNRB	2017	Colorectal	45 N/T	Tissue	Hyper methylation.	Mousavi Ardehaie [55]
signaling pathways						
APC, DDK3, SFRP2, SFRP4, SFRP5, WIF1, WNT5A	2014	Colorectal	125 N/T	Tissue	Aberrant methylations.	Samaei [56]
APC	2009	Esophageal	45 N/T	Tissue	Hyper methylation.	Zare [57]
SFRP2	2016	Colorectal	25 patients 25 controls	Fecal	Hyper methylation.	Babaei [58]
PTEN, miR-21	2016	Colorectal	125 N/T	Tissue	MiR-21 over expression, PTEN under expression.	Yazdani [59]
PTEN	2011	Breast	53 patients 20 controls	Tissue	Hyper methylation.	Sadeq [60]
PTEN	2016	Breast	103 patients 102 controls	Blood	Hyper methylation.	Yari [61]
developmental factors						
ALX4	2015	Colorectal	25 patients 25 controls	Serum	Hyper methylation.	Salehi [62]
PAX5	2018	Gastric	35 patients 35 controls	Blood	Hyper methylation.	Haghverdi [63]
MiR-129-2	2019	Gastric	50 N/T	Tissue	Hyper methylation.	Alizadeh [64]
nuclear receptors						
ER*-a* (ER3,4,5)	2012	Breast	60 patients	Tissue	Hyper methylation.	Ramezani [65]
ER-*a*	2012	Breast	100 patients	Tissue	Hyper methylation.	Izadi [66]
ER-*a*	2012	Breast	49 patients 51 controls	Tissue		Izadi [67]
RARB, p16	2011	Prostate	42 patients 21 controls	Tissue	Hyper methylation.	Ameri [68]
apoptosis						
APAF1,CASP8	2018	Gastric	30 patients 30 controls	Blood	Hyper methylation.	Azarkhazin [69]
FADD, FAS	2014	Oral	86 patients 68 controls	Tissue	FAS promoter hyper methylation.	Saberi [70]
ATM	2015	Brain	30 patients 2 controls	Tissue	Hyper methylation.	Mehdipour [71]
CTLA4	2014	Gastric	85 N/T	Tissue	Hyper methylation.	Kordi-Tamandani [72]

^a^ Tumor tissues and normal margins.

^b^ Malignant tumors.

^c^ Benign tumors

## DNA repair

DNA hyper methylation of tumor suppressor genes have been reported in immortalized and transformed cells [73]. The O6-methylguanine DNA methyltransferase (MGMT) is involved in methylated guanosine repair through removing alkyl group from O6-alkyl guanine [74]. CDH1 as a cell-cell adhesion factor has a critical function in regulation of cell differentiation and normal structure of epithelial cells [75–77]. The MGMT and CDH1 promoter methylations were assessed among a sample of Iranian OSCC patients compared with normal margins. It has been observed that there were CDH1 and MGMT promoter hyper methylations in majority of cases. Moreover, there was a significant difference in MGMT mRNA expression levels between OSCC patients and controls. It was concluded that the MGMT methylation can be used as a proper marker of poor survival among Iranian patients with advanced OSCC [30]. Similarly, there was a significant inverse association between MGMT methylation and survival among a sample of American oral cancer patients, while the frequency of MGMT hyper methylation was noticeably lower than that among Iranian patients [78]. BRCA1 is involved in DNA repair, homologous recombination, and cell cycle regulation [79]. P16 is also a regulator of G1 to S phase during cell cycle progression [80]. Histone modification and DNA methylation of MGMT, BRCA-1, and P16 were assessed in a sample of Iranian breast cancer patients. It has been shown that the promoter methylation of MGMT and BRCA-1 were higher in malignant breast tumor (MBT) compared with benign breast tumor (BBT) cases, while the P16 promoter methylation was lower in MBT patients compared with BBT. There was a significant correlation between BRCA1 hyper methylation and poor survival. Moreover, MBT cases had hypo methylation of histone H4 lysine 20 (H4K20) and hypo acetylation of histone H3 on lysine 18 (H3K18). There was a significant inverse association between H3K9ac levels and tumor size in MBT cases [31]. Similarly, the ratio of MGMT promoter methylation was significantly higher in a sample of Chinese breast cancer patients compared with controls. Moreover, there was a significant converse correlation between MGMT methylation and levels of MGMT protein expression [81]. TP53 encodes a phosphoprotein involving in regulation of apoptosis, cell cycle, DNA repair, and differentiation [82]. It has been observed that there was a significant inverse association between MGMT promoter methylation and P53 expression among a sub population of Iranian glioblastoma patients. They showed MGMT methylation in about half of the patients [32]. P53 is stabilized by posttranslational modification in the primary stages of glioblastoma progression [83]. The MGMT suppression induces p53 mutation which can further deregulate the methylation pattern of MGMT [84]. The role of MGMT promoter methylation in glioblastoma progression was also assessed among German cases and was shown that there was a correlation between MGMT promoter methylation and survival in newly diagnosed patients [85]. It has been observed that there were significantly higher levels of MGMT promoter methylation in tumors compared with controls in a sample of Iranian colorectal cancer (CRC) patients. Moreover, they observed the MGMT promoter methylation in normal margins [33]. Another group also assessed the serum MGMT methylation which showed that the majority of a sample of Iranian CRC tumors had MGMT promoter methylation which were mainly moderately differentiated and located on left colon [34]. Similarly, MGMT promoter methylation has been reported in majority of brain metastases from CRC and corresponding primary tumors in a group of Italian patients [86]. The placenta have also a characteristic of tumor cells for a successful implantation of the embryo in uterus during early pregnancy, in which it invades into the host tissues, escapes from immune response, and promotes angiogenesis. There are similar DNA methylation patterns between the tumors and placenta. The expression profile of the genes located within cancer/placenta hypomethylated blocks were assessed for CRC that showed the epigenetic regulation of NF-kB signaling during tumorigenesis and placentogenesis [87]. Human mutL homolog 1 (hMLH1) is one of the components of mismatch repair (MMR) system that is involved in the replacement of incorrectly paired nucleotides during DNA replication [88]. Therefore, the MMR aberrations can be associated with tumor progression [89, 90]. E-cadherin is a cell adhesion glycoprotein which is related to the tumor metastasis in a hyper methylated status [91]. It has been observed that there was a significant inverse association between the levels of hMLH1 mRNA expression and promoter methylation status in a sample of Iranian gastric cancer patients. Moreover, the hMLH1 hyper methylated tumors were significantly observed in advanced stage tumors. The E-cadherin promoter methylation was also significantly correlated with tumor stage and location [35].

## Cell adhesion

Ras association domain family (RASSF) consists of 10 proteins that act as scaffolding agents in microtubule stability, mitotic cell division, apoptosis, cell migration, cell adhesion, inflammation, and NF-kB regulation [92]. RASSF6 and RASSF10 stabilize P53, regulate the cell cycle, inhibit tumor cell migration, and induce apoptosis [93–97]. Moreover, they are involved in regulation of NF-kB and WNT signaling pathways [93, 98]. Methylation status of RASSF6 and RASSF10 were assessed in a sample of Iranian Acute lymphocytic leukemia (ALL) cases. It was observed that the RASSF6 methylation was more frequent in *B*-Cell Acute Lymphoblastic Leukemia (B-ALL) cases compared with T-cell acute lymphoblastic leukaemia (T-ALL) cases, whereas the RASSF10 hyper methylation was more frequent in T-ALL compared with pre-B-ALL and B-ALL patients. Moreover, there was a significant correlation between RASSF6 hyper methylation and poor prognosis in pre-B-ALL patients which can be related to the NF-kB activation in the absence of RASSF6 [36]. HIC1 is a transcriptional suppressor involved in embryogenesis, P53 dependent apoptosis, cell cycle regulation, and WNT signaling regulation. It has been reported that there were significant correlations between tumor sizes more than 2 cm, lymph node involvement, and HIC1 methylation among a sub population of Iranian breast cancer patients. Moreover, there was a significant association between SASSF1A and HIC1 promoter methylation. It was concluded that the HIC1 and RASSF1A hyper methylations can be used as prognostic markers of breast cancer in this population [37]. Similarly, the RASSF1A methylation has been shown as an efficient prognostic marker in a sample of Saudi breast cancer patients [99]. Thyroid Stimulating Hormone Receptor (TSHR) is involved in growth and function of thyrocytes through stimulation of iodine uptake by NIS and iodine oxidation by thyroid peroxidase [100]. The RARb2 is a thyroid-steroid hormone receptor which is involved in embryogenesis through binding with retinoic acid [101]. It has been reported that there were higher rates of p16, TSHR, and RASSF1A hyper methylations in a sample of Iranian malignant papillary thyroid tumors compared with benign tumors [38]. TSHR methylation status was also introduced as a tumor marker for well-differentiated thyroid cancer among Turkish patients [102]. Integrin α4 binds with integrin β1 and β7 which are associated with cell adhesion to fibronectin [103]. The α4 integrin hyper methylation was observed in the majority of an Iranian prostate cancer patients group [39]. E-cadherin (CDH1) is a trans-membrane glycoprotein mainly expressed on the epithelial cells surface which is involved in Ca2+-dependent intracellular adhesion. CDH1 down regulation is associated with invasiveness and poor prognosis [104–106]. It has been shown that the tumor tissues had higher rates of CDH1 hyper methylation compared with normal samples in Iranian breast cancer patients. Moreover, there were significant associations between CDH1 promoter methylation, stage, grade, lymph node metastasis, and tumor size [40]. Another study on Iranian breast cancer cases also showed a significant higher ratio of CDH1 promoter hyper methylation in tumors compared with normal tissues [41]. The SPG20 is a multifunctional protein involved in intracellular EGFR traffic, cytokinesis, lipid droplet turnover, bone morphogenetic protein (BMP) signaling inhibition, and E3 ubiquitin ligases regulation [107–111]. It has been observed that the percentage of methylated reference (PMR) values in plasma samples of CRC patients were significantly higher than that in the healthy individuals among a sub population of Iranian subjects. The receiver-operating characteristics (ROC) curve analysis showed a sensitivity of 81.1% which was significantly higher than carcinoembryonic antigen (CEA) tumor marker (48.6%). Therefore, plasma SPG20 promoter methylation status can be an efficient noninvasive biomarker for CRC among Iranians [42].

## Cell cycle

**Fig. 2:**
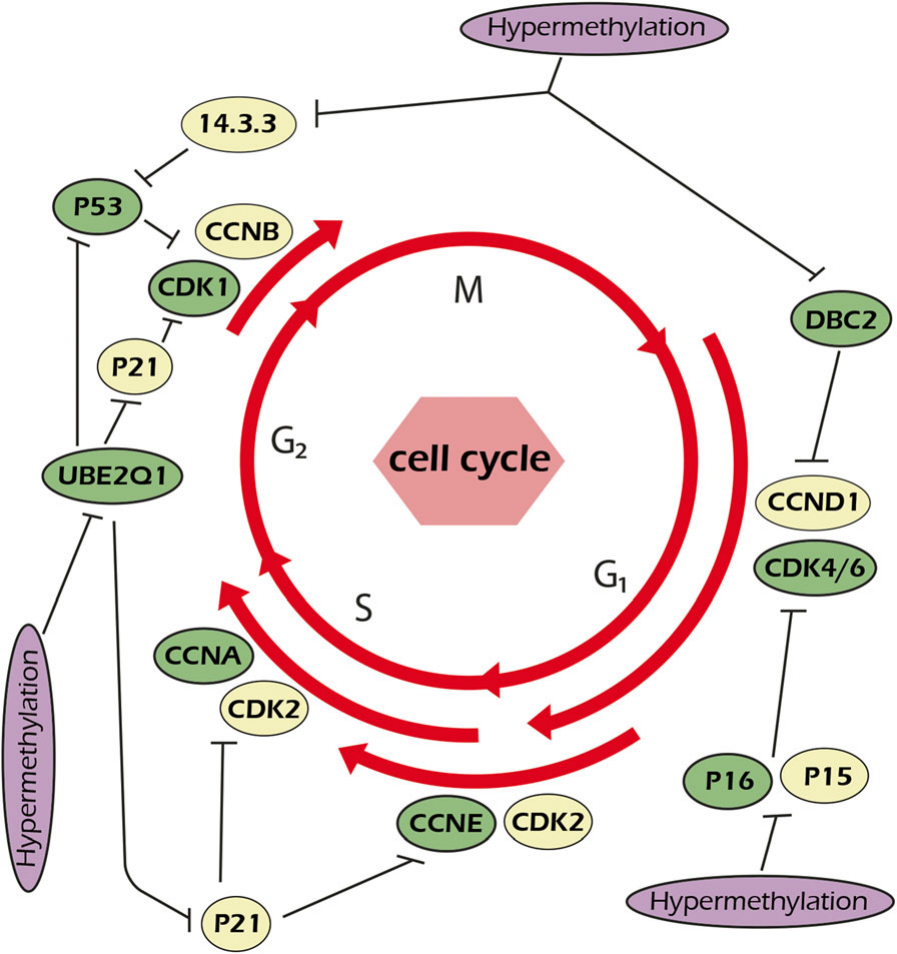
aberrant methylation of cell cycle regulators during tumor progressions among Iranian patients

P14ARF is a cell cycle regulator that inhibits the MDM-2 mediated degradation of p53 [112–114]. It has been reported that there was higher ratio of p14ARF methylation in a sample of Iranian oral squamous cell carcinoma (OSCC) patients compared with controls which was also directly correlated with tumor stage [43]. Similarly, It was reported that there were significant associations between p14ARF hyper methylation, advanced stages, and lymph node involvement among Japanese OSCC patients [115]. The p53, p16INK4a, and MDM2 have critical roles during cell cycle regulation [116]. MDM2 is an oncogene that inactivates p53 during tumorigenesis [117, 118]. The G1 to S cell cycle progression is regulated by CCND1 in relationship with CDK 4/6 which is suppressed by the inhibitor of cyclin dependent kinase 4 (INK4) [119]. The INK4 family includes p19INK4D, p15INK4B, p18INK4C, and p16INK4A [112]. It has been shown that the p16INK4A inhibits G0/G1 cell cycle through suppression of CCND1–CDK4/6 complex. The p16INK4A also functions as a negative regulator of retinoblastoma protein (Rb) and regulates the cell cycle in G1/S phase in a p53-dependent pathway through CDK4 and CDK6 inhibitions [120, 121]. It has been observed that there was a significant inverse correlation between p16 hyper methylation and P53 expression in a sample of Iranian *esophageal* squamous cell *carcinoma* (ESCC) patients [44]. The serum p16 promoter hyper methylation was associated with poor prognosis among Japanese ESCC patients [122]. Another study on Iranian subjects assessed the p16 methylation status between familial and sporadic ESCC cases compared with healthy subjects. It was shown that the sporadic cases had higher ratio of p16 methylation compared with familial ESCC cases, while there was not any p16 methylation among controls [45]. Similarly, p16 methylation rate in sporadic was higher than that in familial Korean colorectal cancer patients [123]. Another group has been reported that there were direct correlations between p16 hyper methylation, tumor grade, HP infection, and smoking in a subpopulation of Iranian OSCC cases [46]. Another group has been reported that there was higher ratio of p16 and p15 methylations in tumors compared with normal margins in a sample of Iranian OSCC patients [47]. The aberrant methylation of the p15 and p16 have been also reported during OSCC progression among Japanese patients [115]. The p16 promoter hyper methylation was also involved in primary stages of sporadic breast cancer in Iranian patients [48]. Another study on Iranian gastric cancer patients showed that the P16 hyper methylation was less frequent in well-differentiated tumors and more frequent in older patients [49]. DBC2 is a tumor suppressor gene that functions through down-regulation of CCND1 [124]. It is also involved in regulation of ubiquitination, cell cycle, protein transport, apoptosis, and cytoskeleton [125–128]. It has been reported that there was significantly higher frequency of DBC2 methylation in tumor and blood samples of a group of Iranian breast cancer patients compared with normal margins [50]. Similarly, a study on Chinese breast cancer patients showed higher DBC2 methylation in breast tumors compared with normal tissues. Moreover, there was a significant correlation between DBC2 promoter methylation and lymph node metastasis [129]. The 14-3-3σ is a p53-related G2/M suppressor associated with DNA repair and apoptosis [130, 131]. It has been reported that there was higher ratio of 14-3-3σ promoter methylation in a sample of Iranian breast tumors compared with normal tissues [51]. Similarly, 14-3-3σ promoter methylation was higher in Chinese breast tumors compared with benign and normal tissues [132]. Ubiquitin-proteasome system (UPS) has a critical role in cell cycle regulation [133, 134]. The protein modification by ubiquitin is an important strategy for the elimination of abnormal proteins. UPS is also associated with pathophysiological processes during tumor progression [133]. Ubiquitination is performed by ubiquitin-activating enzymes (E1), ubiquitin-conjugating enzymes (E2), and ubiquitin-protein ligases (E3). UBE2Q2 and UBE2Q1 are members of E2 ubiquitin-conjugating enzyme family [135]. UBE2Q2 functions as an oncogene during CRC initiation and progression [136, 137]. It has been observed that there were higher levels of methylated UBE2Q1 in colorectal tumor samples compared with normal margins among a sub population of Iranian subjects [52]. Aberrant methylation of cell cycle regulators during tumor progressions among Iranian patients are illustrated in Fig. 2.

## Tyrosine kinase and G-protein-coupled receptors

The downstream of tyrosine kinase type 7 (DOK7) is an adaptor protein that induces the acetylcholine receptors (AChR) through muscle-specific kinase (MUSK) [138, 139]. It also inhibits proliferation and migration of cancer cells via AKT signaling pathway [140]. The vimentin is an intermediate filament that plays important roles in epithelial mesenchymal transition (EMT), immune response, and cytoskeleton structure [141–144]. CXCR4 (C-X-C chemokine receptor type 4) is a receptor involved in calcium signaling, transcription, chemotaxis, cell survival, and proliferation. The CXCR4 promoter hypo methylation has been detected in melanoma, breast, and pancreatic cancers [145, 146]. SAM pointed domain containing ETS transcription factor (SPEDF) is a tumor suppressor involved in tumor progression via p21/CIP1 regulation [147, 148]. It has been reported that there were significant DOK7, VIM, and CXCR4 hypo methylations in a sub population of Iranian breast cancer cases compared with normal subjects. Moreover, there were significant correlations between DOK7 and VIM methylations and negative ER status [53]. Another reports also showed DOK7 and VIM hyper methylations in Spanish and Australian breast cancer patients, respectively [140, 149]. The growth hormone secretagogue receptor (GHSR) belongs to the G-protein-coupled receptor (GPCR) family which acts as a receptor for ghrelin [150]. Ghrelin is associated with regulation of glucose and lipid metabolism and activates Ca2+ and P13K/AKT signaling pathways that are contributed with secretion of growth hormone in pituitary cells [151–153]. It has been reported that there was significant hyper methylation of GHSR in a sample of Iranian gastric cancer tissues compared with normal margins [54]. Similarly, GHSR hyper methylation was also observed in Italian colorectal cancer tissues compared with normal tissues [154]. Endothelin receptor type B (EDNRB) is a G protein coupled receptor involved in embryonic and enteric ganglia development [155–157]. Decreased expression of EDNRB leads to proliferation, angiogenesis, and metastasis through ET1 signaling pathway during tumor progression [158–160]. KISS1R is also a G-protein coupled receptor that is associated with tumor metastasis by ERK inhibition and MMP-9 reduction [161, 162]. It has been reported that there was higher frequency of EDNRB hyper methylation in a sample of Iranian colorectal cancer tissues compared with normal margins [55]. Similarly, the Chinese colorectal cancer tumors had significantly higher frequency of EDNRB promoter hyper methylation compared with normal tissues [155].

## Signaling pathways

The WNT signaling pathway is involved in embryogenesis and tumor progression [163–165]. DNA methylation of APC, AXIN2, SFRP, and DKK as important WNT inhibitors have been reported in colorectal cancer patients [166–169]. It has been observed that there were significant correlations between APC and DDK3 aberrant promoter methylations and age and sex, respectively among a sub population of Iranian colorectal patients. The SFRP4 and WIF1 promoter methylations were significantly associated with stage and grade. Moreover, there were significant correlations between SFRP2 and SFRP5 methylations and tumor type. Univariate analysis also indicated the WIF1 promoter methylation as a prognostic factor in colorectal cancer patients [56]. Adenomatous polyposis coli (APC) is a tumor suppressor involved in regulation of cell growth through WNT signaling. In normal cells, free β-catenin is phosphorylated by Axin-APC-GSK3β complex which results in β-catenin proteasomal degradation and reduced expression of WNT signaling target genes [170]. It has been reported that there were higher rates of APC hyper methylation in tumor tissues compared with normal tissues in a sample of Iranian ESCC patients. Moreover, the hyper methylated cases had lower survival rates. There was also a direct association between APC promoter hyper methylation and grade of tumor differentiation [57]. The Chinese esophageal cancer tumors had also higher rates of APC methylation compared with controls [171]. SFRP2 is one of the negative regulators of WNT signaling pathway. It has been observed that there were higher levels of SFRP2 hyper methylation in a sample of Iranian CRC patients compared with healthy subjects [58]. Similarly, there were also high levels of SFRP1 and SFRP2 hyper methylations among a group of Hungarian CRC patients [172]. Phosphatase and tensin homolog (PTEN) is a suppressor of PI3K/AKT pathways which inhibits signal transduction from HER1, HER2, and IGFR growth factor receptors through the P13K/AKT signaling [173, 174]. It forms a nuclear complex with p53 to inhibit the p53 decomposition [175, 176]. Moreover, it induces G0-G1 cell cycle arrest by suppression of CCND1 and ERK/MAPK pathway [177]. MiR-21 promotes tumor cell growth and invasion by PTEN targeting [178–180]. It has been reported that there was a significant association between PTEN promoter methylation and expression in a sample of Iranian colorectal cancer patients. The levels of PTEN mRNA expressions were inversely associated with miR-21 expression. Moreover, there were converse significant associations between PTEN expression, tumor size, survival, and tumor stage [59]. Similarly, it has been observed that there was a significant correlation between PTEN promoter methylation and expression among sporadic Indian breast cancer patients [181]. Another study on Iranian sporadic breast cancer patients showed that there were correlations between PTEN hyper methylation, advanced stages, and lymph node involvement. They suggested the PTEN promoter methylation as a prognostic marker for the response to PTEN-dependent therapy [60]. Iranian Kurdish breast cancer patients also had a higher frequency of PTEN methylation compared with healthy controls. The female relatives of patients had also a significantly higher frequency of PTEN methylation compared with controls. Moreover, the PTEN methylation was higher in patients between 40-80 years old compared with patients who were between 29–39 years old which showed increased PTEN methylation in higher ages [61].

## Developmental factors

Homeobox protein aristaless-like (ALX4) is a homeodomain transcription factor associated with bone, skin, and hair follicle development [182, 183]. It has been reported that there was a significant difference of ALX4 methylation status between a sample of Iranian colorectal cancer patients and controls which introduced that as an efficient marker for the early detection of colorectal cancer in this population [62]. Similarly, ALX4 methylation was observed among German patients with colorectal, esophageal, and gastric cancers [184]. Paired Box 5 (PAX5) is belonged to the PAX family of tissue-specific transcription factors associated with development and embryogenesis. Deregulation of PAX5 has been observed in various types of human tumors [185]. It is involved in neoplastic transformation through CD19 regulation which suppresses growth regulators [186]. Moreover, PAX5 is a functional tumor-suppressor in liver carcinogenesis by P53 regulation [187]. Methylation status of PAX5 was assessed in blood samples of Iranian gastric cancer patients compared with healthy blood samples. There were higher levels of PAX5 methylation in the blood samples of patients compared with controls. There were also significant correlations between the mean expression levels of PAX5, age, and promoter methylation status [63]. It has been shown that there was a significant correlation between PAX5 methylation and survival in a sample of Chinese gastric cancer patients [188]. MicroRNAs (miRNAs) are one of the main factors in gene regulation in normal and tumor tissues which function through 3’ un-translated region (3'UTR) dependent translational inhibition [189–192]. The miRNAs expressions are regulated by methylation, alkylation, and acetylation [193–195]. MiR-192-2 induces the apoptosis through targeting SOX4 in gastric tumor cells [196]. It has been shown that there was a significant difference of miR-129-2 methylation between a sample of Iranian gastric cancer and healthy cases [64].

## Nuclear receptors

Estrogen and its receptors are involved in breast epithelial cell homeostasis through regulation of proliferation, differentiation, and apoptosis. The methylation of estrogen receptors including ER-*α* and ER*-β* play important role in primary breast cancer progression. Loss of ER-*α* is an important mechanism of hormone resistance in breast cancer [197–201]. It has been observed that there was significantly higher ER4 methylation in tumors with P53 expression among a sub population of Iranian breast cancer patients. The ER5 methylation was observed in tumors with lymph node metastasis and higher grades. ER4 and ER5 methylations in postmenopausal females were higher than that in premenopausal cases. There was also significant higher frequency of ER5 methylation in Her-2+ tumors. ER-α hyper methylation was frequently observed in invasive ductal cell carcinoma patients. Moreover, there was a direct correlation between ER5 methylation and age [65]. ER-*α* promoter methylation status in Egyptian breast cancer patients were assessed using MSP method which showed higher ratio of methylation in ER3 and ER5 compared with Iranian patients [202]. Another study assessed the ER-α methylation among Iranian breast cancer patients which showed methylation in majority of basal and Her2+ tumors. There was a correlation between ER-α methylation and poor prognosis in basal and Her2+ tumors. They showed that the ER-α methylation plays an important role in aggressive breast tumors in this population [66]. ER3 and ER5 methylations have been also reported in majority of a sample of Iranian ER negative breast tumors [67]. Retinoic acid receptor beta (RARB) belongs to the thyroid-steroid hormone receptors which bind with retinoic acid to mediate embryogenesis and cell differentiation. It has been reported that there was higher frequency of RARB hyper methylation in poor prognosis cases compared with good prognosis in a sample of Iranian prostate cancer patients. The p16 hyper methylation in poor prognostic cases was also higher than patients with good prognosis [68]. Similarly, RARB methylation was associated with a higher prostate cancer risk among American patients [203].

## Apoptosis

**Fig. 3:**
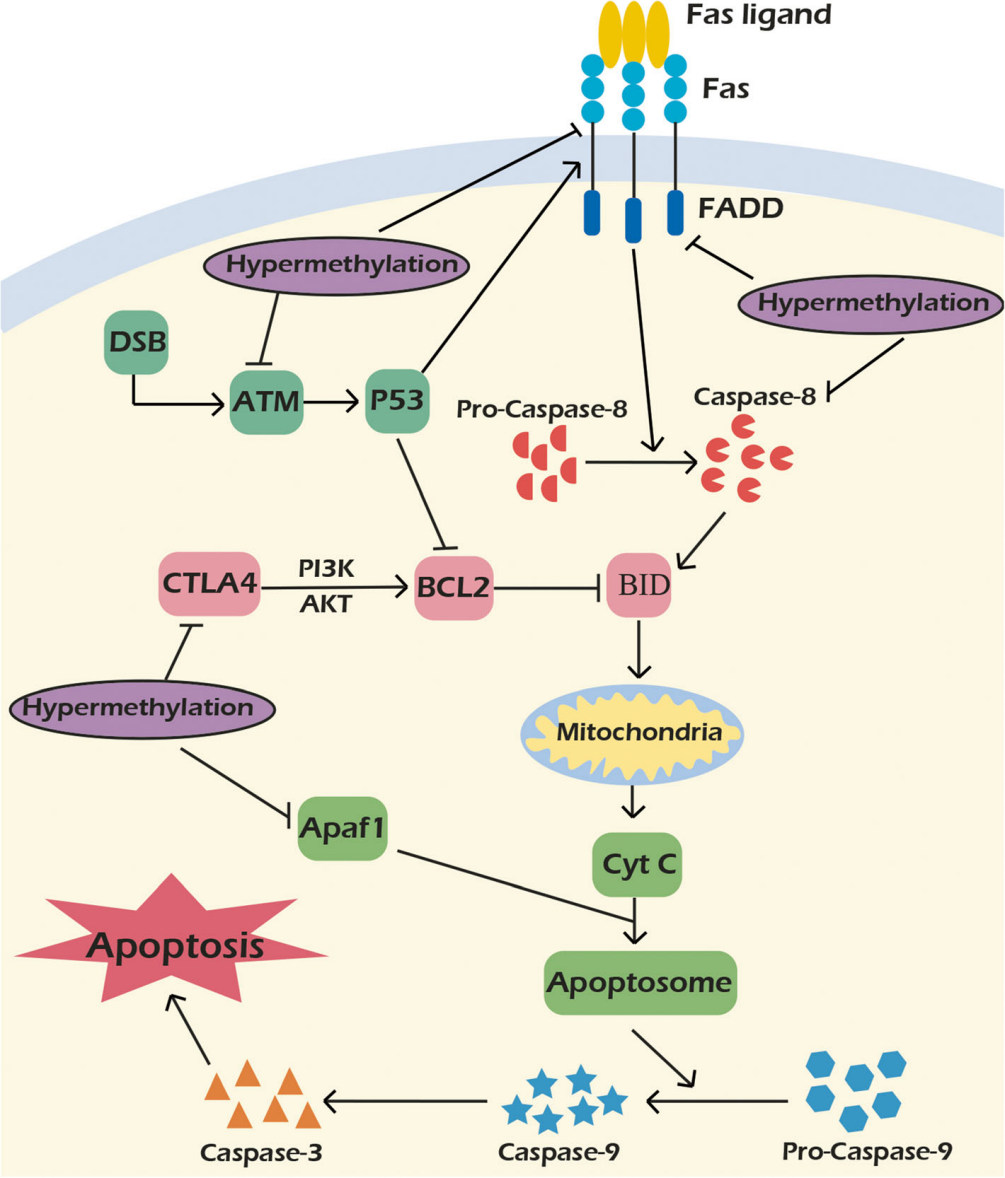
role of aberrant methylation in regulation of apoptosis during tumor progressions among Iranian patients

The apoptotic protease activating factor 1 (APAF1) and caspase 8 (CASP8) genes are important regulators of apoptotic pathways. Extrinsic apoptosis pathway is mediated by CD95, FADD, and procaspase-8 in which the CASP8 triggers the proteolytic activation of other caspases and cleavage of cellular substrates [204–209]. Cytochrome c is released from the mitochondria following DNA damage and binds to the APAF1 in cytosol that results in CASP9, CASP3, CASP6, and CASP7 serial activations and apoptosis [210, 211]. It has been shown that there was a significant association between the levels of APAF1 methylation, tumor stage, and grade in blood samples of a subpopulation of Iranian gastric cancer patients. Moreover, the CASP8 methylation status in blood samples of patients was significantly correlated with age [69]. Chinese gastric cancer patients had also significant higher ratio of APAF1 methylation in their tumor tissues compared with normal margins [212]. Fas belongs to the tumor necrosis factor receptor (TNF-R) family that is normally expressed in lymph nodes and spleen [213]. Fas Ligand (FasL) acts as a ligand for Fas receptor that activates CASP8 through Fas-associated death domain (FADD). Subsequently, CASP8 activates CASP3 and CASP7 that mediate cell death. Moreover, it cleaves BID to generate truncated BID which enters to the mitochondria and triggers the mitochondrial apoptotic pathway [214, 215]. It has been observed that there was aberrant FAS promoter methylation in majority of a sample of Iranian oral squamous cell carcinoma patients, whereas the aberrant FADD methylation was observed in a minority of cases [70]. Ataxia telangiectasia mutated (ATM) is a serine threonine kinase which is activated by DNA double-strand break (DSB). Deregulated expression of E2F1 transcription factor up regulates ATM that leads to the apoptosis induction, cell cycle regulation, and DNA repair via phosphorylation of CHK1, CHK2, P53, and CDC25 [216, 217]. ATM promoter methylation was more frequent in meningioma and glioma patients in a sample of Iranian cases. There was a significant correlation between higher grades of brain tumors and ATM promoter methylation. There was also a significant association between ATM promoter methylation and RB expression. Moreover, there was a significant association between D1853N polymorphism and ATM promoter methylation [71]. Another report assessed the promoter methylation status of ATM among Indian breast cancer cases which showed significant higher ratio of promoter hyper methylation in tumor tissues compared with normal samples. Moreover, there were significant correlations between ATM promoter methylation, age, tumor size, and advanced tumor stages [218]. Cytotoxic T-lymphocyte-associated antigen-4 (CTLA4) is a receptor that acts as an immune check point in regulation of immune responses and is expressed on activated T-cells [219, 220]. It induces the PKB/AKT activation which up regulates the BCL-XL/BCL-2 [221]. It has been observed that there was significantly higher frequency of CTLA4 promoter methylation in a sample of Iranian gastric cancer patients compared with normal margins [72]. In contrast, a study on Qatari breast cancer patients showed significant hypo methylation of CpG islands in promoter region of CTLA-4 in tumors compared with normal margins [222]. Role of aberrant methylations in regulation of apoptosis during tumor progressions among Iranian patients are illustrated in Fig. 3.

## Conclusions

Regarding the recent life style changes, there is a growing cancer incidence and mortality in Iran which is related to the late diagnosis. Epigenetic markers are considered as emerging diagnostic and prognostic biomarkers in cancer. Therefore, in present review we summarized all of the methylation abnormalities during tumor progressions which have been reported until now among Iranian patients. It was frequently observed that the p16 and CDH1 aberrant promoter methylations can be involved in tumor progression of ESCC, thyroid, oral, breast, gastric, and prostate cancers. The MGMT promoter hyper methylation was also frequently reported in CRC, GB, BC, and OSCC. Therefore, p16, CDH1, and MGMT methylation status can be suggested as a general methylation based panel marker for all cancers in Iranian patients. Moreover, there were various reports of PTEN and ER-α promoter hyper methylations in Iranian BC patients which introduces them as methylation based markers of BC in this population. Generally, this review paves the way to introduce a non-invasive methylation specific panel of diagnostic markers for the early detection of cancer among Iranian populations.

## Data Availability

The datasets used and/or analyzed during the current study are available from the corresponding author on reasonable request.

## Abbreviations

DNMT: DNA methyltransferase
HATs: Histone acetyl-transferase
HMTs: Histone methyltransferase
MBT: Malignant breast tumor
BBT: Benign breast tumor
CDH1: E-cadherin
BMP: Bone morphogenetic protein
PMR: Percentage of methylated reference
ROC: Receiver-operating characteristics
CEA: Carcinoembryonic antigen
Rb: Retinoblastoma protein
UPS: Ubiquitin-proteasome system
DOK7: Downstream of tyrosine kinase type 7
AChR: Acetylcholine receptors
EMT: Epithelial mesenchymal transition
SPEDF: SAM pointed domain containing ETS transcription factor
GHSR: Growth hormone secretagogue receptor
GPCR: G-protein-coupled receptor
PTEN: Phosphatase and tensin homolog
miRNAs: MicroRNAs
3'UTR: 3’ un-translated region
RARB: Retinoic acid receptor beta
FasL: Fas Ligand
TNF-R: Tumor necrosis factor receptor
ATM: Ataxia telangiectasia mutated
DSB: Double-strand break
CTLA4: Cytotoxic T-lymphocyte-associated antigen-4
OSCC: Oral squamous cell carcinoma
MGMT: O6-methylguanine DNA methyltransferase
MLH1: MutL homolog 1
BRCA1: Breast cancer type 1 susceptibility protein
H4K20: Histone H4 lysine 20
H3K18: Histone H3 on lysine 18
MMR: Mismatch repair
ALL: Acute lymphocytic leukemia
B-ALL: *B*-Cell Acute Lymphoblastic Leukemia
T-ALL: T-cell acute lymphoblastic leukaemia
TSHR: Thyroid Stimulating Hormone Receptor
RASSF: Ras association domain family
ESCC: *Esophageal* squamous cell *carcinoma*
EDNRB: Endothelin receptor type B
ALX4: Homeobox protein aristaless-like
PAX5: Paired Box 5
APAF1: Apoptotic protease activating factor 1
CASP8: Caspase 8
CRC: Colorectal cancer
hMLH1: Human mutL homolog 1
